# The CCL2/CCR2 Axis Affects Transmigration and Proliferation but Not Resistance to Chemotherapy of Acute Myeloid Leukemia Cells

**DOI:** 10.1371/journal.pone.0168888

**Published:** 2017-01-03

**Authors:** Patricia Macanas-Pirard, Thomas Quezada, Leonardo Navarrete, Richard Broekhuizen, Andrea Leisewitz, Bruno Nervi, Pablo A. Ramírez

**Affiliations:** 1 Hematology Oncology Department, School of Medicine, Pontificia Universidad Católica de Chile, Santiago, Chile; 2 Institute of Medicine, Faculty of Medicine, Universidad Austral de Chile, Valdivia, Región de los Ríos, Chile; Katholieke Universiteit Leuven Rega Institute for Medical Research, BELGIUM

## Abstract

Acute myeloid leukemia (AML) has a high mortality rate despite chemotherapy and transplantation. Both CXCR4/SDF-1 and VLA-4/VCAM1 axes are involved in leukemia protection but little is known about the role of CCL2/CCR2 in AML biology and protection against chemotherapy. We measured CCR2 expression in AML cell lines and primary AML cells by flow cytometry (FCM), real time PCR (RT-PCR) and western blot (WB). CCL2 production was quantified by solid phase ELISA in peripheral blood (PB) and bone marrow (BM) serum. We measured chemotaxis in a transwell system with different concentrations of CCL2/CCR2 blockers; cell cycle with BrDU and propidium iodide and proliferation with yellow tetrazolium MTT. We determined synergy in *in vitro* cell apoptosis combining chemotherapy and CCL2/CCR2 blockade. Finally, we performed chemoprotection studies in an *in vivo* mouse model. Of 35 patients, 23 (65%) expressed CCR2 by FCM in PB. Two cell lines expressed high levels of CCR2 (THP-1 and murine AML). RT-PCR and WB confirmed CCR2 production. CCL2 solid phase ELISA showed significantly lower levels of CCL2 in PB and BM compared to normal controls. Chemotaxis experiments confirmed a dose-dependent migration in AML primary cells expressing CCR2 and THP-1 cells. A significant inhibition of transmigration was seen after CCL2/CCR2 blockade. Proliferation of CCR2+ AML cell lines was slightly increased (1.4-fold) after axis stimulation. We observed a non-significant increase in phase S THP-1 cells exposed to CCL2 and a concomitant decrease of cells in G1. The chemotherapy studies did not show a protective effect of CCL2 on cytarabine-induced apoptosis or synergy with chemotherapy after CCL2/CCR2 blockade both *in vitro* and *in vivo*. In conclusion, CCL2/CCR2 axis is expressed in the majority of monocytoid AML blasts. The axis is involved in cell trafficking and proliferation but no *in vitro* and *in vivo* chemotherapy protective effect was seen.

## Introduction

Acute myeloid leukemia (AML) is a complex disease with an elevated mortality rate despite high intensity therapies [1]. The mechanisms of resistance and relapse of AML are related to a number of factors [2]. Among them, the interaction between AML and its microenvironment determines resistance against chemotherapy [2, 3]. Multiple receptors and soluble factors are likely involved in this resistance but the way in which they interact is still unclear. Among the better characterized receptors are CXCR4 and VLA4 [3, 4]. However, little is known about the role of CCL2/CCR2 axis in AML biology and protection against chemotherapy.

CCL2 belongs to the family of ß-chemokines [5]. Its gene is located on chromosome 17q11.2 [6], and its main function is associated with the initiation of chemotaxis and transendothelial migration of monocytes [7]. CCL2 expression is regulated by multiple factors. [8]. Upon binding to its receptor, CCL2 activates multiple transduction pathways related to survival, adhesion, cellular mobility, proliferation, growth and protein transduction [9].

The role of CCL2/CCR2 axis in cancer is largely unknown. In *in vivo* solid tumor models (breast, gastric and ovarian cancers), it was shown that CCL2/CCR2 axis mediated the migration of MSC into the tumor and also showed evidence of CCL2-mediated protumor effect. CCR2 -/- mice had attenuated tumor growth compared to wild-type mice [10]. In human AML samples, it was shown that CCR2 was almost exclusively expressed on monocytoid AML [11]. Interestingly, CCL2 expression and production showed high levels mostly in monocytoid blasts [11]. In another series however, CCL2 levels were significantly lower in the subgroup of monocytoid M4 and M5 AML patients [12]. In FIP1L1-PDGFRA+ eosinophilic leukemia expressing CCR2, it was shown that CCL2 induced cell chemotaxis and strong migration involving GCPR, PKC, PLC, p38 MAPK and NF-κB [13].

In this study we show in a series of experiments with both AML cell lines and primary AML cells an important role of CCL2/CCR2 axis in AML cell trafficking and proliferation but not in protection against chemotherapy.

## Materials and Methods

### In vivo studies

#### Mice

C57BL/6J and 129Sv/J mice were obtained from the Jackson Laboratory (Bar Harbor, ME, USA). The mCG^PR/+^ strain has been previously described and was maintained on a C57BL/6 × 129/SvJ F1 background [14]. Hybrid C57BL/6J x 129Sv/JF1 (B6129F1) mice at 9 to 18 weeks of age were used in all the experiments. Animal care and euthanasia protocols were approved by the Bioethics and Biosafety Commission of the Faculty of Biological Sciences, Pontificia Universidad Católica de Chile (approval ID: CBB-2008). Briefly, mice were euthanized by an overdose of anesthesia (Ketamine/Xylazine 300 mg/Kg and 30mg/Kg respectively) by an intraperitoneal injection.

#### Acute promyelocytic leukemia cells and transplantation

Acute promyelocytic leukemia cells (APL) from the spleens of mCG-PML-RAR knock in mice (B6129F1) were harvested and cryopreserved [14]. APL cells (10^6^ cells/mouse) were injected intravenously via the tail vein into genetically compatible B6129F1 recipients, without pre-treatment with any radiation or chemotherapy conditioning.

#### Mobilization protocol and treatments

Plerixafor (AMD3100) (Genzyme, Cambridge, MA) was supplied as a sterile isotonic aqueous solution at 20 mg/ml and was administered at a dose of 5 mg/Kg as a subcutaneous injection. The CCR2 antagonist, SC202525 (Santa Cruz Biotechnology, Dallas, TX) was supplied as a sterile lyophilized powder, soluble in DMSO (100 mM). SC202525 was administered at a dose of 2 mg/Kg as a subcutaneous injection.

#### Colony-forming cell assay (CFC)

Colony-forming cell (CFC) or colony-forming unit (CFU) assays were performed by plating blood in methylcellulose medium for mouse cells supplemented with recombinant cytokines (MethoCult GF 3434; Stem Cell Technologies, Vancouver, BC Canada) according to standard techniques [15]. The assay is based on the ability of hematopoietic progenitors to proliferate and differentiate into colonies in a semi-solid media in response to cytokine stimulation. The colonies formed can be enumerated and characterized according to their unique morphology. This aims to study differences in the percentage of leukemia CFU/total APL number plated.

#### Blood counts and flow cytometry

To ensure leukemia development and engraftment, peripheral blood (PB) samples were taken from the tail of mice for complete blood counts using an automated cell counter (Sysmex KX-21N, Sysmex America, Inc., Mundelein, IL) and flow cytometry (FCM) (BD FACS Canto II, BD Biosciences, San Diego, CA). The spleens from dead or euthanized animals were analyzed for evidence of AML. Single-cell suspensions from PB samples were stained with fluorescein isothiocyanate (FITC)-conjugated anti-mouse CD117, phycoerythrin (PE)-conjugated anti-mouse CD34 and allophycocyanin (APC)-conjugated anti-mouse Ly-6G and Ly-6C (myeloid differentiation antigen, Gr-1) (all from BD Biosciences Pharmingen, San Diego, CA). A minimum of 10,000 events were acquired for each sample by FCM and data analyzed using FACSDiva software (BD Biosciences, San Diego, CA).

### In vitro studies

#### Reagents

Cytarabine (Ara-C) was purchased from Pfizer (Bentley, WA, Australia). Recombinant human CCL2, CCL8 and CCL7 were purchased from R&D Systems (Minneapolis, MN, USA).

#### Cell culture and purification

The murine leukemia APL cells (myeloid origin) were kindly provided by Dr. John DiPersio (Washington University School of Medicine, St. Louis, USA). The AML cell lines U-937 (monocytoid), THP-1 (monocytoid) and Kasumi (M2), were purchased from ATCC (Manassas, VA). All cell lines (murine and human) were cultured in RPMI 1640 (Invitrogen, Carlsbad, CA, USA), supplemented with 10% (v/v) FBS, 100 IU/ml penicillin and 100 μg/ml streptomycin, non-essential amino acids and 2 mM L-glutamine in a humidified incubator at 37°C with 5% carbon dioxide.

Primary AML samples from PB or bone marrow (BM) were obtained from patients with newly diagnosed *de novo* AML after written informed consent in accordance with institutional guidelines set forth by the Catholic University of Chile and before chemotherapy treatment. This study was reviewed and approved by the Catholic University of Chile School of Medicine Institutional Ethics Committee before samples were taken. BM aspirates collected during standard diagnostic procedures were obtained from the posterior iliac crest and collected in a heparinized syringe. Mononuclear cells were purified by Ficoll-Hypaque density-gradient centrifugation as described by the manufacturers (GE Healthcare Bio-sciences AB, Uppsala, Sweden). Blast purity exceeded 90%. Primary cells were resuspended in RPMI 1640 medium supplemented with 10% (v/v) FBS, 100 IU/ml penicillin and 100 μg/ml streptomycin, non-essential amino acids and 2 mM L-glutamine in a humidified incubator at 37°C with 5% carbon dioxide. **Table 1**shows the general diagnostic laboratory data for the 35 AML patients in the study. The percentage of leukemia blasts corresponds to the amount of blasts present in whole BM or PB samples, before gradient separation.

**Table 1:** AML Patients included in the study (n = 35).

Patient	Sex	% CCR2 BM	%CCR2 PB	Sample	Used for experiments
**1**	**M**	**12**	**9**	**BM / PB**	**no**
**2**	**F**	**2.6**	**2**	**BM / PB**	**no**
**3**	**M**	**-**	**1.7**	**PB**	**no**
**4**	**M**	**63**	**45**	**BM / PB**	**no**
**5**	**F**	**1**	**3.5**	**BM/PB**	**no**
**6**	**F**	**2.6**	**1.2**	**BM/PB**	**no**
**7**	**F**	**1.3**	**4.8**	**BM/PB**	**no**
**8**	**F**	**27**	**22**	**BM/PB**	**no**
**9**	**M**	**6**	**6**	**BM/PB**	**yes**
**10**	**F**	**-**	**7.5**	**PB**	**no**
**11**	**M**	**-**	**19**	**PB**	**no**
**12**	**M**	**15**	**14**	**BM/PB**	**yes**
**13**	**M**	**16**	**6**	**BM/PB**	**yes**
**14**	**F**	**14**	**0**	**BM/PB**	**no**
**15**	**F**	**3**	**1.5**	**BM/PB**	**no**
**16**	**F**	**3**	**-**	**BM**	yes
**17**	**M**	**5.3**	**5.3**	**BM/PB**	**no**
**18**	**M**	**3.5**	**-**	**BM**	**no**
**19**	**M**	**8**	**6**	**PB/BM**	**no**
**20**	**M**	**0.1**	**-**	**BM**	**no**
**21**	**M**	**15**	**10**	**BM**	yes
**22**	**M**	**9**	**-**	**BM**	**no**
**23**	**F**	**3.3**	**-**	**BM**	**no**
**24**	**M**	**1**	**-**	**BM**	**yes**
**25**	**M**	**2.6**	**-**	**BM**	**no**
**26**	**M**	**8**	**7**	**BM/PB**	yes
**27**	**F**	**22**	**14**	**BM/PB**	yes
**28**	**M**	**15**	**-**	**BM**	**no**
**29**	**M**	**9**	**-**	**BM**	yes
**30**	**F**	**7.5**	**13.4**	**BM/PB**	yes
**31**	**F**	**11**	**-**	**BM**	yes
**32**	**M**	**55**	**53**	**BM/PB**	yes
**33**	**M**	**8.6**	**-**	**BM**	yes
**34**	**F**	**5**	**-**	**BM**	yes
**35**	**M**	**90**	**90**	**BM**	yes

**M,** Male; **F**, Female; **BM,** bone marrow; **PB**, peripheral blood.

#### Flow cytometry analysis of CCR2 expression

For CCR2 expression studies, AML cell lines, BM or PB samples were stained at room temperature with saturating concentrations of phycoerythrin-conjugated anti-CCR2, fluorescein isothiocyanate-conjugated anti-CD14, peridinin chlorophyll–conjugated CD45 and allophycocyanin-conjugated anti-CD34/Kit monoclonal antibodies for 1 hour. Samples were washed once with 5ml BD FACS lysing solution (BD biosciences, San Jose, CA USA) and washed again with 5ml phosphate buffered saline and then analyzed by FCM. We considered positive for CCR2 expression all AML samples with FCM readings above 2% as determined by data from all screened patients and respective isotype controls.

#### Flow cytometry analysis of cell proliferation

THP-1 cells were seeded on 96-well plates and treated with various concentrations of recombinant human CCL2 (10, 50 and 100 ng/ml) for 24 hours before cultures were harvested and analyzed by FCM. The amount of cells in each sample was counted for 50 seconds.

#### Treatment of AML cells

THP-1 cells cultured in RPMI 1640 were incubated with recombinant human CCL2 (10 ng/ml) or a mixture of recombinant human CCL8 (50 mg/ml), CCL7 (50 mg/ml) and CCL2 (10 mg/ml) for at least 2 hours before treatment with Ara-C (1, 5 and 20 μg/ml). AML cells were exposed to Ara-C for 24 hours before analysis of cell viability or apoptosis.

#### Cell migration assay

Migration of THP-1 and primary AML cells from 3 patients with the highest CCR2 expression by flow cytometry was assayed using the Transwell system (3-μm pores, Corning, Pittsburg, PA, USA) as previously described [16]. THP-1 (3x 10^5^ cells/well), U-937 (3x 10^5^ cells/well) or primary AML cells (8x 10^5^ cells/well) grown in RPMI supplemented with 2% FBS, were placed in the upper chamber (500 μl) exposed to different doses of recombinant chemokine CCL2 (optimum dose 10 ng/ml) placed in the lower chamber. Cell cultures were incubated and allowed to migrate overnight (15 hours) at 37°C with 5% carbon dioxide and subsequently cells from both chambers were harvested and counted by FCM analysis for 50 seconds/sample and also counted using a Neubauer hemocytometer to determine transmigration. Results are expressed as the percentage migration relative to control (spontaneous) migration. THP-1 and U-937 migration to cytokine SDF-1 was included as a positive control. SDF-1 is the ligand for CXCR4, a pivotal receptor involved in AML cell trafficking and mobilization. For migration-inhibition experiments, THP-1 or primary AML cells were pre-incubated for 30 minutes with a blocking human CCR2 monoclonal antibody (10 μg/ml) (R&D systems) or CCR2 receptor antagonist, sc202525 (5 nM). In some experiments, cell migration was assayed in the presence of neutralizing monoclonal antibody against CCL2 (5 μg/ml) (R&D systems). Isotype-matched antibodies were used as negative controls.

#### Detection of cell viability

THP-1 cells were cultured in 96-well plates for Ara-C treatment for 24 hours. Cell viability was assessed by the MTT (3-(4,5-dimethylthiazol-2-yl)-2,5-diphenyl tetrazolium bromide) assay (Sigma, St. Louis, MO). Two hours before ending the 24-hour treatment, 10 μl of MTT (5 mg/ml saline) was added to each well, and the samples were incubated for 2 hours at 37°C. Cells were lysed and MTT crystals solubilized by the addition of 100 μl of 0.02 N HCl in isopropanol. The absorbance of each well was determined at 590 nm using a BioTek microplate reader (BioTek instruments, Winooski, VT). Cell viability (%) was calculated relative to the control.

#### Annexin V staining

THP-1 cell apoptosis was assessed by FCM using the annexin V-FITC apoptosis detection kit as described by the manufacturers (BD Biosciences Pharmingen, San Diego, CA). In short, THP-1 cells were grown in 24-well plates before treatment with Ara-C for 24 hours. Cultures were harvested and washed once in phosphate-buffered saline (PBS) and resuspended in 1X binding buffer in PBS with 1% BSA and incubated with Annexin V-FITC and APC anti-mouse Ly-6G and Ly-6C (Gr-1) (BD Pharmingen) for 30 minutes. Cells were then washed once with PBS and resuspended in 1X binding buffer in PBS supplemented with PI. All data was acquired on a BD FACSCanto II cytometer and analyzed using FACSDiva software (BD Biosciences)).

#### Cell cycle distribution

THP-1 cells grown in 24-well plates with or without recombinant human CCL2 (50 mg/ml) for 24 hours were incubated with bromodeoxyuridine at various time-points as described by the manufacturers (FITC BrdU flow kit, BD Pharmingen, San Diego, CA). THP1 cells were harvested, fixed and labelled with anti-BrdU fluorescein isothiocyanate antibody and 7-AAD to analyze cell cycle status by FCM.

#### Protein extraction and Western blot

Cell pellets were washed twice in ice cold PBS and lysed with lysis buffer and protease inhibitors (20 mM Tris (pH 7.5), 1% triton, 10% glycerol, 137 mM NaCl and 2 mM EDTA, 250 μM PMSF, 5μg/ml leupeptin) for 20 mins, followed by centrifugation at 10,000 rpm for 10 min at 4°C. Protein concentration of the supernatant was measured using Bio-Rad protein assay dye reagent (Bio-Rad, Hercules, CA, USA). Fifty micrograms of proteins were equally loaded to a 10% SDS-polyacrylamide gel, electrophoresed, and transferred to polyvinylidene difluoride membrane (PVDF) (Thermo Scientific, Rockford, IL, USA). Membranes were developed using Pierce^®^ ECL Western blotting substrate (Thermo Scientific). The following anti-human antibodies were used for immunoblotting: CCR2 and β-actin (Cell Signaling Technology).

#### Isolation of RNA and qRT-PCR

Total RNA was extracted using Trizol (Invitrogen) as described by the manufacturer. Complementary DNA was subsequently synthesized from total cellular RNA using MMLV reverse transcriptase (Promega, Madison, WI, USA). Real-time PCR (qRT-PCR) was performed using fast SYBR^®^ green master mix (Applied Biosystems, Hercules, CA, USA) according to the manufacturer’s instructions. qRT-PCR was carried out with a StepOne Applied Biosystems thermal cycler. All the reagents used were from Applied Biosystems. Reactions were run in triplicate and the genes of interest were normalized to glyceraldehyde-3P-dehydrogenase (GAPDH). The primers sequences were as follows: CCR2 forward primer: TGACAGGCACAGATGAATGG; CCR2 reverse primer: ATCATCTCCTGGCTGAATGC; GAPDH forward primer: CACCCAGAAGACTGTGGATGG; GAPDH reverse primer: CCACCAC-CCTGTTGCTGTAG.

#### Solid phase enzyme-linked immunosorbent assay (ELISA)

BM and PB samples from AML patients and healthy donors were centrifuged for 5 mins at 2,000 rpm room temperature to obtain the blood plasma. Plasma samples were processed to quantify the amount of autocrine CCL2 (ligand for CCR2 receptor) production by solid phase enzyme-linked immunosorbent assay (ELISA) using the human CCL2 quantikine ELISA kit (R&D Systems). The assay was followed according to the manufacturer’s instructions.

#### Statistical analysis

All data is given as means ± S.D. of at least three independent experiments. Statistical analysis of data was made using one-way analysis of variance (ANOVA) followed by Bonferroni`s least significant difference post hoc test. Statistical analysis was performed using GraphPad Prism 5 statistical package. The significance level chosen for the statistical analysis was ^*^P < 0.*05*.

## Results

### CCR2 expression on AML cell lines and primary AML cells

**Fig 1:**
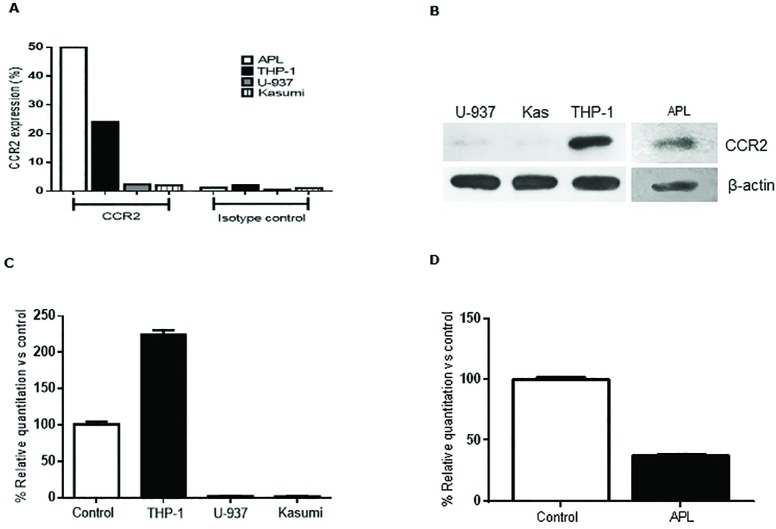
CCR2 expression in human and murine AML cell lines. Human AML cells lines THP1, U-937 and Kasumi and murine AML cell line (APL) were cultured to detect CCR2 expression by FCM (**A**), WB (**B**) and qPCR (**C and D**). All cell lines were grown in RPMI medium and were processed for the various studies according to the material and methods section. Positive control samples correspond to PB or BM samples from healthy individuals with 5–8% normal monocytes. Healthy monocytes show above 90% CCR2 expression.

To evaluate the role of CCL2 /CCR2 in AML biology, we first determined CCR2 expression in human AML cell lines (THP-1, U-937 and Kasumi-1) and the murine AML cell line (APL) by flow cytometry (FCM), western blot (WB) and real time PCR (qPCR). FCM analysis with CCR2 MoAb demonstrated high surface expression of CCR2 in THP-1 and APL cells lines, whereas low CCR2 expression was observed with human AML cell lines U-937 and Kasumi (Fig 1A). Similarly, positive chemokine CCR2 receptor expression was confirmed for THP-1 and APL cells by WB, whereas weak bands for CCR2 were observed for U-937 and Kasumi cell lines (Fig 1B). qPCR analysis of AML cell lines with specific primers for CCR2 also showed the presence of CCR2 transcripts in both THP-1 and APL cells compared to the positive control (human or murine healthy PB samples) (Fig 1C and 1D). Positive controls correspond to PB or BM samples from healthy patients with 5–8% normal monocytes. Healthy monocytes show above 90% CCR2 expression (data not shown). Altogether this data demonstrated that human and murine AML cell lines, THP-1 and APL express the receptor CCR2.

**Fig 2:**
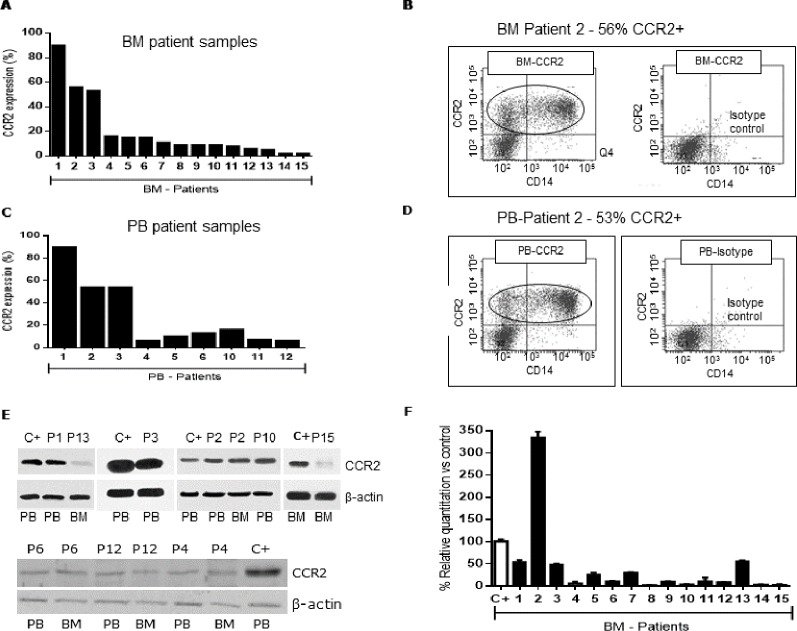
Expression of CCR2 by AML patient samples. (**A**) Leukemia blasts purified from BM primary samples were analyzed for CCR2 expression by FCM. (**B**) Patient 2 BM FCM analysis (histogram) is shown as an example with 56% CCR2+ leukemia cells. (**C**) Leukemia blasts purified from PB primary samples were analyzed for CCR2 expression by FCM. (**D**) Patient 2 PB FCM analysis (histogram) is shown as an example with 53% CCR2+ leukemia cells. (**F**) Overall analysis of CCR2 expression profile by qPCR from all BM patient samples compared to positive control. Positive control sample corresponds to a BM sample from a healthy donor with 5% normal monocytes.

We processed 35 newly diagnosed *de novo* AML patients (median age: 55.4 years, range 16–89) and 6 healthy donors (with informed consent). We found 23/35 AML patients (65%) had CCR2 expression compared to their isotype controls. Mean CCR2 expression was 19% (range 3.5–90%) in PB and 20% (range 3–90%) in BM. Fig 2A shows the percentage CCR2 expression in 15 BM AML patient samples analyzed by FCM with positive CCR2 expression (Table 1). From the 15 BM AML patient samples, we had available for some patients PB samples which were also analyzed for CCR2 expression by FCM (Fig 2C). Fig 2B (BM sample) and 2D (PB sample) show FCM histograms of patient 2 analyzed for CCR2 expression. The histograms show a large population of positive CCR2 leukemia blasts (surrounded in black), 56% (BM) and 53% (PB).

To further confirm our findings on CCR2 expression in these AML patient samples, we detected CCR2 receptor expression by WB for patients in which we had enough material for protein purification. Fig 2E shows CCR2 expression in 6 BM and 7 PB patient samples. There were bands observed for CCR2 receptor protein expression (42kDa) for all analyzed patient samples (both PB and BM samples) (Fig 2E). These patients also showed positive CCR2 expression by FCM (Fig 1A and 1C). qPCRs were performed on 28/35 (80%) of our AML patient samples to quantify mRNA levels for CCR2 expression. qPCR data of AML patient samples showed that 85% of patients have mRNA expression for CCR2. Fig 2F shows the relative quantification of CCR2 mRNA profile expression relative to the positive control (BM sample from a healthy donor), which confirms expression of CCR2 transcripts in all 15 patient samples. In particular, patient 2 showed the highest mRNA level expression for CCR2 in BM samples (Fig 2F). Similarly, this patient showed high expression of CCR2 receptor in BM and PB samples confirmed by FCM (BM sample-56% CCR2 expression, Fig 2B). Overall as shown in Fig 2, we did not observe a direct correlation between CCR2 expression by qPCR vs protein expression by WB or FCM. Some patients had elevated qPCR signal but low protein quantity by WB.

### CCL2/CCR2 axis on AML cell trafficking and proliferation

**Fig 3:**
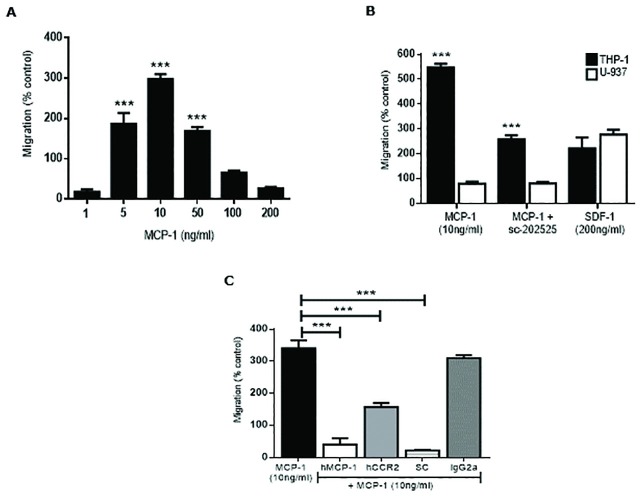
THP-1 cell migration in response to CCL2. (**A**) THP-1 cells were seeded on the upper chamber of a transwell plate and incubated with various concentrations of CCL2 (1, 5, 10, 50, 100 and 200 ng/ml) in the lower chamber overnight to measure THP-1 cell migration. (**B**) THP-1 (CCR2+) and U-937 (CCR2-) cell migration study was performed by exposing leukemia cells to CCL2 (10 ng/ml), sc-202525 (5 nM) (CCR2 inhibitor) or SDF-1 (200 ng/ml) (CXCR4 inhibitor, positive control) overnight. (**C**) THP-1 cells were exposed to CCL2 (10 ng/ml), anti-CCL2 (5 μg/ml), anti-CCR2 (10 μg/ml), sc-202525 (5 nM) and isotype control antibody overnight. Leukemia cell migration for each study was measured by FCM. Each bar represents the mean ± SD of 3 independent experiments (***p < 0.001).

To explore the possible role of CCL2/CCR2 axis on AML cell trafficking, we subsequently determined whether the incubation of THP-1 and primary AML cells with human recombinant CCL2 could stimulate AML cell migration in vitro. Migration of THP-1 and primary AML cells was determined as described in Materials and Methods. We used a transwell system with permeable supports with 3μm pores. We first exposed THP-1 cells placed on the upper chamber to various concentrations of CCL2 placed in the lower compartments of the migration system. As shown in Fig 3A, the migration response of THP-1 cells was significantly enhanced with CCL2 doses of 5, 10 and 50 ng/ml compared to 1, 100 or 200 ng/ml. THP-1 transmigration reached a plateau at 10 ng/ml leading to lower levels of cell migration observed with CCL2 50, 100 and 200 ng/ml. CCL2 10 ng/ml is the optimum dose of cell migration in THP-1 cells. To confirm these findings, we performed a migration study of THP-1 (CCR2 +) and U-937 cell line (CCR2 -) to compare migration. Fig 3B shows significant transmigration of THP-1 cells compared to U-937 with no migration (migration below 100% is considered background) with CCL2 (10 ng/ml). THP-1 migration was significantly reduced by treatment with the small molecule CCR2 inhibitor, sc-202525. THP-1 and U-937 showed positive migration to cytokine SDF-1 included as a positive control. SDF-1 is the ligand for CXCR4, a pivotal receptor involved in cell trafficking and mobilization in hematopoietic cells. The CXCR4 receptor is expressed in the THP-1 and U-937 cells and has traditionally been considered the standard for chemotaxis experiments [17–19]. As observed in Fig 3C, migration of THP-1 cells was significantly reduced in the presence of a neutralizing CCL2 monoclonal antibody and also by a blocking CCR2 monoclonal antibody. Furthermore, almost complete inhibition in THP-1 transmigration was detected after CCL2/CCR2 blockade by antagonist sc-202525 (Fig 3C). The isotype control (IST) to the monoclonal antibodies showed no change in THP-1 cell migration. The small molecule inhibitor showed no toxicity to the THP-1 cells (data not shown).

**Fig 4:**
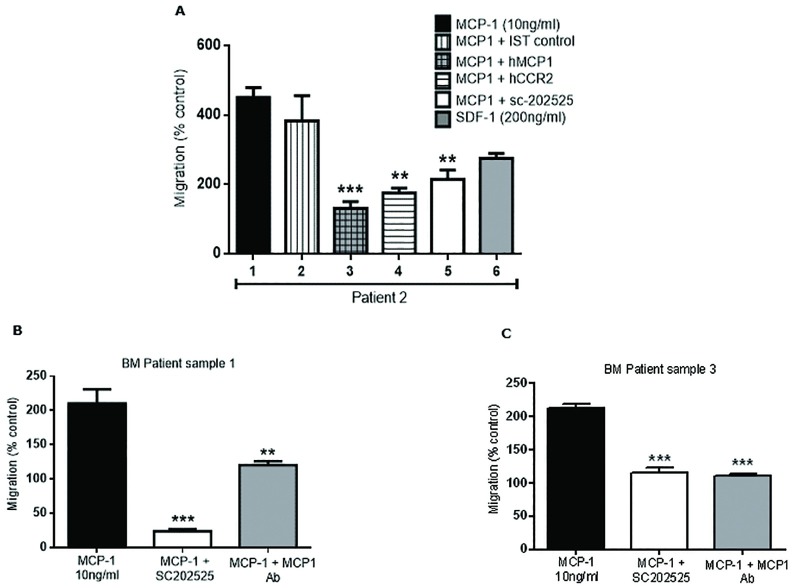
Cell migration analysis of primary AML cells in response to CCL2. (**A**) Primary purified leukemia cells (Patient 2—Table 1) were seeded on the upper chamber of a transwell plate and incubated with CCL2 (10 ng/ml), isotype control antibody, anti-CCL2 (5 μg/ml), anti-CCR2 (10 μg/ml), sc-202525 (CCR2 inhibitor, 5 nM) or SDF-1 (200 ng/ml) (positive control) overnight to measure leukemia cell migration. Patient 1 (CCR2+, Table 1) (**B**) and Patient 3 (CCR2+, Table 1) (**C**) leukemia cell migration was also measured in response to CCL2, sc-202525 or anti-CCL2 incubation overnight. Primary leukemia cell migration for each study was measured by FCM. Each bar represents the mean ± SD of 3 independent experiments (**p < 0.01; ***p < 0.001).

We then performed migration studies with patient AML samples. Fig 4A shows significant transmigration of primary AML cells-patient 2 (which expresses abundant CCR2 as confirmed by FCM-Fig 2) to the effects of CCL2 (10 ng/ml) (Fig 4A). A significant inhibition on transmigration was observed after monoclonal antibodies against CCL2 or CCR2 were used. Similarly, migration was significantly reduced by CCL2/CCR2 blockade with a small molecule inhibitor against CCR2, sc-202525. The IST to the monoclonal antibodies to CCL2 or CCR2 showed no change in AML cell migration. SDF-1 (positive control) showed a 2-fold increased migration over controls. Fig 4B and 4C shows positive migration of primary AML cells from patient 1 and patient 3 respectively. Both patients showed high CCR2 expression by FCM (Fig 2). Leukemia cell migration was significantly reduced using a monoclonal antibody against CCL2 or the CCR2 inhibitor sc-202525. Overall, both AML primary cells and cell lines that lacked CCR2 expression failed to transmigrate under the effect of CCL2. This data confirms that the CCL2/CCR2 axis shows functional activity and plays a role in AML blast migration.

**Fig 5:**
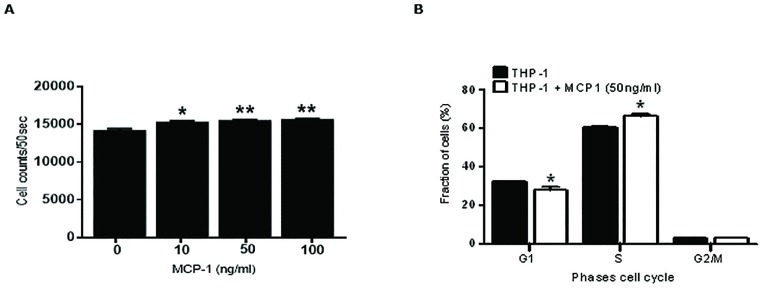
Cell proliferation and cell cycle analysis of THP-1 cells exposed to CCL2. (**A**) THP-1 cells were seeded on a 96-well plate and incubated with various concentrations of recombinant human CCL2 (10, 50 and 100 ng/ml) for 24 hours and cell proliferation measured by FCM. (**B**) THP-1 cells were cultured with or without CCL2 (50 ng/ml) for 24 hours. Leukemia cells were harvested, fixed and stained with PI and analyzed by FCM. Each bar represents the mean ± SD of 3 independent experiments (*p < 0.05; **p < 0.01).

We also investigated whether the CCL2/CCR2 axis shows an effect on AML cell proliferation and cell cycle when exposed to CCL2. First, the THP-1 cells were incubated with various concentrations of CCL2 (10, 50 and 100 ng/ml) for 24 hours and the data was analyzed by FCM. As observed in Fig 5A, THP-1 cells showed a significant increase in cell proliferation (1.4-fold) compared to controls after the exposure to CCL2. This data was also confirmed using the MTT viability assay (data not shown).

We have also optimized the bromo-deoxyuridine (BrdU) labeling technique to investigate THP-1 cell cycle analysis. THP-1 cells were pre-incubated for 4 hours with CCL2 (50 ng/ml) before staining with BrdU. Cells were allowed to proliferate overnight before FCM analysis. Interestingly, data showed a slight increase in S phase (DNA synthesis) of THP-1 cells with CCL2 treatment (50ng/ml) compared to THP-1 control (Fig 5B). Furthermore, this slight increase in S phase was associated with a decrease of THP-1 cells in G1 phase. This data confirmed that cytokine CCL2 stimulates THP-1 cells to proliferate.

We also performed both proliferation and migration studies with the APL cells. Migration studies showed significantly lower APL migration than THP-1 cells (data not shown). Proliferation studies with CCR2+ cell lines THP-1 and APL, and CCR2- cell line U937 showed that both CCR2+ cell lines proliferated significantly more after exposure to CCL2 compared to CCR2- cell line U937 (data not shown).

### Effect of CCL2 on chemotherapy induced apoptosis in THP-1 cells

**Fig 6:**
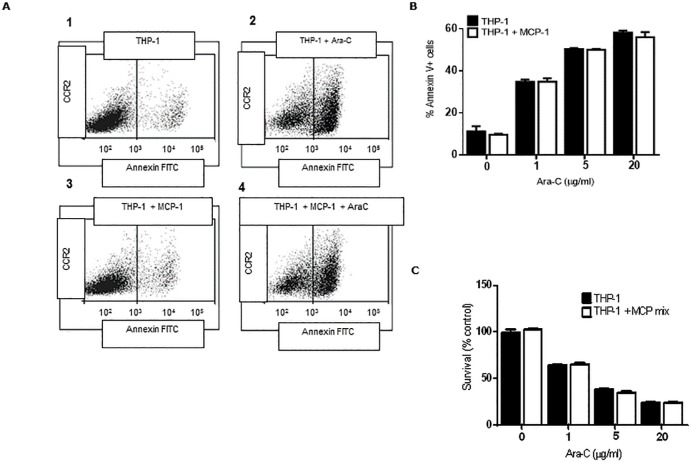
In vitro chemosensitivity analysis of THP-1 cells to Ara-C. (**A**) Representative histograms of healthy (1 and 3) and apoptotic populations (2 and 4) of THP-1 cells incubated with and without CCL2 (10 ng/ml) for 2 hours before treatment with Ara-C (20 μg/ml) for 24 hours for the analysis of cell death by apoptosis by FCM. The annexin V positive apoptotic populations are highlighted in black. Overall chemosensitivity studies of THP-1 cells incubated with and without CCL2 (10 ng/ml) (**B**) or CCL2, -2 and -3 cocktail mix (**C**) for 2 hours before treatment with Ara-C (1, 5, 20 μg/ml) for 24 hours and cell viability measured by the MTT assay. Each bar represents the mean ± SD of 3 independent experiments.

We previously published that the BM stromal cells secrete soluble factors that protect human AML cells from Ara-C induced apoptosis [20]. We explored whether cytokine CCL2, normally secreted by BM stromal cells, could chemoprotect THP-1 cells from Ara-C induced apoptosis. THP-1 cells were incubated with and without human recombinant CCL2 (10 ng/ml) for 2 hours before treatment with Ara-C (1, 5 and 20 μg/ml) for 24 hours. Apoptosis was measured by FCM using the Annexin V-FITC apoptosis kit. Fig 6A shows representative histograms of healthy (histograms 1 and 3) and apoptotic populations of THP-1 cells with and without exposure to CCL2 and treatment with Ara-C (histograms 2 and 4). The annexin V positive apoptotic populations are highlighted in black. Data revealed a dose-dependent increase in THP-1 cell apoptosis by Ara-C treatment. However, data demonstrated a non-protective effect on THP-1 cells from apoptosis by CCL2 treatment (Fig 6B). Higher doses of CCL2 treatment (50, 100 and 200 ng/ml) showed no effect on chemoprotection of THP-1 cells (data not shown). We have also evaluated chemoprotection studies using THP-1 cells with the combination of chemokines (human recombinant CCL2, MCP-2 and MCP-3). Fig 6C shows THP-1 cells alone (control) or incubated with CCL2, MCP-2 and MCP-3 before treatment with Ara-C for 24 hours. Cell survival was measured by MTT. Similarly, THP-1 cells showed no chemoprotection by exposure to the MCPs from the effects of Ara-C. Overall, these in vitro studies show that the CCL2/CCR2 axis plays an important role in AML cell trafficking and proliferation, although no in vitro chemotherapy protective role was observed.

### Quantification of CCL2 levels in plasma samples from AML patients

**Fig 7:**
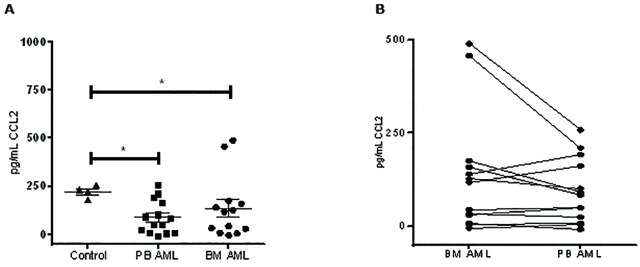
Quantification of CCL2 in plasma samples from AML patients. (**A**) Plasma from BM and PB samples from AML and healthy patients was processed for CCL2 quantification by solid phase ELISA. (**B**) Correlation of CCL2 detection between BM and PB samples from each patient. Each bar represents the mean ± SD of 3 independent replicates (*p < 0.05; **p < 0.01).

We performed a solid phase ELISA with PB and BM blood plasma samples from 18 recruited patient samples (AML patients and healthy donors) to quantify the amount of autocrine CCL2 production. As observed in Fig 7A, significant higher levels of CCL2 in healthy donors (223.3 pg/ml CCL2) compared to AML patients was seen (PB 89.6 pg/ml CCL2, BM 138 pg/ml CCL2). No significant difference in CCL2 levels was observed between BM and PB AML samples. Fig 7B shows the correlation of CCL2 between BM and PB within the same patient. A slight tendency of higher CCL2 in the BM samples compared to the PB samples for each patient can be observed. However, no significant correlation was found between groups. Overall, the data show there is lower quantity of CCL2 in AML patients compared to healthy donors.

### In vivo AML model, the impact of CCL2/CCR2 axis blockade in AML survival and progression

We previously demonstrated in vivo that the BM stroma supports and protects AML cells as a mechanism from chemotherapy resistance [20]. It is well known that the SDF-1/CXCR4 axis plays a crucial role in homing AML and HSCs to the BM. Furthermore, we have shown that the CXCR4 inhibitor, AMD3100, mobilizes AML cells from the BM to the PB [3, 20]. We showed that AMD3100 treatment of leukemic mice significantly enhanced the efficacy of chemotherapy (Ara-C) treatment due to AML mobilization from the BM to the PB.

**Fig 8:**
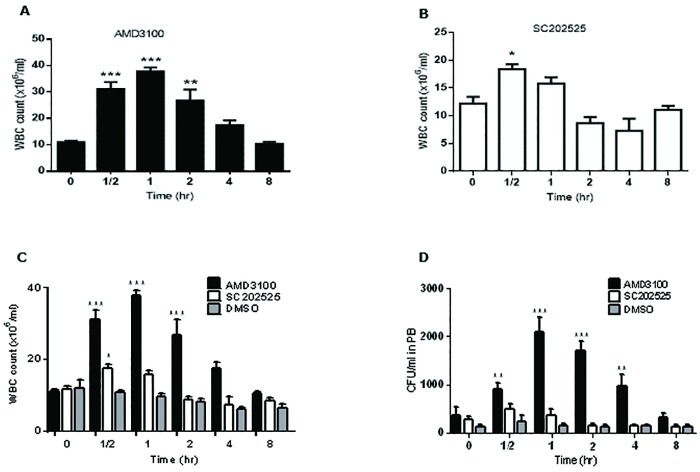
BM CCR2 axis blockade by sc-202525 induced a rapid WBC mobilization. Healthy syngeneic B6129F1 recipient mice were subcutaneously injected with AMD3100 (CXCR4 inhibitor, positive control, 5 mg/Kg, n = 4), sc-202525 (2 mg/Kg, n = 4) or vehicle control (DMSO, n = 4), and PB samples taken at regular intervals of 30 min, 1, 2, 4 and 8 hours post-treatment for WBC count (**A, B and C**) and CFU assay (**D**). Each bar represents the mean ± SD of 4 independent replicates (*p < 0.05; **p < 0.01; ***p < 0.001).

We then investigated whether the CCL2/CCR2 axis plays a role in vivo in AML mobilization and homing. For these in vivo studies, healthy B6 x 129 F1 mice (n = 16) were used to explore WBC mobilization using the CCR2 antagonist, sc-202525 (2 mg/Kg). Healthy mice were subcutaneously injected with the CXCR4 inhibitor, AMD3100 (positive control, 5 mg/Kg) (n = 4), sc-202525 (2 mg/Kg) (n = 4) or with DMSO only (vehicle control group) (n = 4). PB samples were taken at 30 min, 1, 2, 4, 8 hours post-treatment. At each time point, we performed WBC counts and CFU assays to study the mobilization of WBCs and HPCs from the BM to the PB. As observed in Fig 8A, the treatment of healthy mice with AMD3100 resulted in a rapid mobilization of WBCs with peak levels of WBCs at 1 hour post-injection of AMD3100. WBC mobilization was transient as WBC counts returned to baseline levels at 8 hours post-injection. On the other hand, the treatment of mice with the CCR2 inhibitor, sc-202525 also showed a significant increase in WBC mobilization with a peak level at 30 minutes post-injection (Fig 8B). WBC baseline levels returned to normal levels within 8 hours post-injection. The overall effect of CCR2 blockade by sc-202525 showed a modest effect of WBC mobilization compared to AMD3100, although sc-202525 treatment showed a similar pattern of mobilization (Fig 8C). DMSO treatment as a vehicle control showed a slight transient decrease in WBC levels, which again returned to normal baseline levels within 8 hours post-injection (Fig 8C). DMSO treatment (50 μl DMSO) showed no toxicity to healthy mice. The WBC mobilization from BM to PB was confirmed by the CFU assay that revealed a peak level of CFU (stem cell colonies) at 1 hour post-treatment with AMD3100 (Fig 8D). The highest number of stem cell colonies obtained with sc-202525 treatment was also at the 30 minutes post-treatment time-point. This in vivo study suggested that in vivo CCR2 blockade could support AML mobilization.

**Fig 9:**
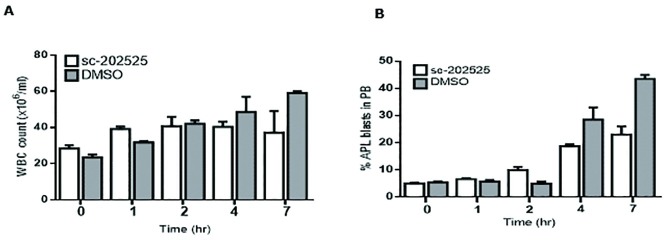
In vivo leukemia mobilization by sc-202525. Syngeneic B6129F1 recipient mice were injected intravenously with 2x 10^6^ APL cells (leukemic). Nine days after APL injection, mice were treated with a single subcutaneous dose of sc-202525 (2 mg/Kg, n = 5) or vehicle control (DMSO, n = 5). PB samples were taken at 0, 1, 2, 4 and 7 hours post-treatment for WBC count (**A**) and FCM analysis for leukemia cell quantification (**B**). Each bar represents the mean ± SD of 5 independent replicates.

Next, we evaluated whether in vivo CCR2 inhibition could cause AML mobilization from the BM to the PB. For this, healthy syngeneic B6 x 129 F1 recipient mice (n = 10) were intravenously injected with 2x10^6^ APL cells. To ensure a minimum percentage of circulating APL cells, eight days after APL injection, mice were screened for leukemia engraftment and trafficking by FCM and WBC count. All mice presented comparable numbers of circulating WBCs and APL blasts (less than 5%, time-point 0 hours (pre-treatment)) in PB and mice were separated into 2 groups. The following day, mice were treated with a subcutaneous injection of sc-202525 (2 mg/Kg) (n = 5) or DMSO treatment (control; n = 5). PB samples were taken at 0, 1, 2, 4 and 7 hours post-treatment for WBC count and FACS analysis. All mice in the sc-202525 treatment group showed an increase in WBCs (Fig 9A). However, within 7 hours post-treatment, all mice died of leukemia with 20% APL cells in PB. Similarly, the WBC counts from mice treated with DMSO showed a fast increase in WBC mobilization up to 4 hours post-treatment, which led to a massive mobilization of leukemia cells from the BM to PB. This resulted in leukostasis and death of all mice within 7 hours post-treatment, with levels of leukemia cells in PB up to 45% (Fig 9B). Due to the toxic effects of DMSO to the leukemic mice, we could not draw any conclusions in vivo as to whether the inhibition of the CCR2 receptor plays a role in AML mobilization and disease progression in our murine AML mouse model.

## Discussion

Despite steady improvements in AML therapy over recent years, AML is still a highly deadly disease, with only 25% of adults surviving 5 years post-diagnosis [21]. Most AML treatments were developed more than 30 years ago, relying mostly on high intensity chemotherapy and allogeneic stem cell transplantation. Still, a large proportion of patients continue to relapse and die after the initial diagnosis and treatment.

Great progress in cellular and molecular biology has allowed us to better understand the process of AML development and evolution [22, 23]. However, this progress has not translated into better treatments against AML.

Surface molecules are potential targets for novel therapies against AML. For example, a clear role in AML biology of CXCR4 receptor has been shown. In *in vivo* experiments and human trials, CXCR4 blockade has demonstrated survival improvement of AML mice models [3, 24] and induced high response rates in relapsed AML patients [25].

Little is known about CCL2/CCR2 axis in AML biology. Recently it has been demonstrated in a series of *in vitro* experiments that upon co-culture of mesenchymal stromal cells (MSC) with AML cells, there was a significant increase of MSC-derived CCL2 secretion [26].

In our series, high expression of CCR2 was observed in AML cell lines and 65% of human AML samples (exclusively monocytoid AML) detected by FCM, WB and qPCR. Several series using human AML samples have shown similar results in CCR2 expression, mostly detected by FCM [11, 27]. Quantification of mRNA levels by quantitative PCR showed that the majority of patients expressed CCR2 mRNA with a big range of variability. This data confirmed previous information showing that CCR2 expression is mostly seen in more differenciated monocytoid AML, which is expected since normally monocytes express high levels of CCR2 related to their function. Interestingly, CCR2 by FCM showed similar levels of expression between BM and PB blasts.

Overall, we observed that our patients had significantly lower CCL2 plasma levels than normal controls. In contrast to our data, Manzur et al [12] showed in their cohort that AML patients presented significantly higher CCL2 levels compared to normal controls. However, their monocytoid patients, similarly to our data, had lower CCL2 levels. It is not clear why, in patients with monocytic leukemias, lower plasma levels of CCL2 or its relevance were observed. One explanation could be downregulation of its secretion or uptake by the receptor in patients with increased CCR2 expression [28]. Bruserud et al [29] showed that primary cells from a cohort of AML patients expressed a broad spectrum of autocrine chemokines involved in chemotaxis, and CCL2 was among the CCL chemokines highly produced by AML primary cells.

To determine CCR2 functionality, we performed a number of experiments to evaluate AML cell trafficking. We showed that under the exposure to CCL2 (CCR2 ligand), a significant dose-dependent transwell migration was seen in cells expressing CCR2, but not in those not expressing the CCR2 receptor. Furthermore, after blockade with a synthetic CCR2 inhibitor (SC202525) as well as monoclonal antibodies against CCL2 and CCR2, a significant inhibition in transwell migration was also observed. As expected, none of the cells lacking CCR2 expression showed transmigration or inhibition of transmigration. Similar results were observed with cell lines and primary AML cells. These results suggest that CCL2/CCR2 axis presents functional activity in AML blast migration in vitro. A recent analysis also confirmed that CCL2/CCR2 axis is relevant in AML blast migration using a human AML cell line (THP-1) and an *in vivo* model [30].

We also sought to analyze if CCL2/CCR2 axis was involved in AML proliferation. Previous literature has shown that CCL2 is involved in cell proliferation of different cell lines [31–33]. In relation to AML, there is little information about its role on AML proliferation and cell cycle. We observed a slight, but statistically significant, increase in cell proliferation after the exposure to increasingly high concentrations of CCL2. This was also associated with a significant decrease in G1 phase and an increase in S phase. These results suggest that *in vitro*, CCL2 stimulates CCR2+ cells to proliferate. However, the numbers were very small and its relevance is unclear. Bruserud et al. [29] showed that exogenous CCL2 on growth factor dependent proliferation was variable in several AML primary cells expressing CCR2 similar to our findings where a small effect on proliferation was observed. It has been extensively shown that the microenvironment protects tumor cells against chemotherapy effects [3, 20, 34]. In our experiments we did not observe significant *in vitro* chemoprotective effect of CCL2 and other MCPs after exposing AML cells to cytarabine. It has not been previously published if CCR2-CCL2 axis is involved in the microenvironment protection against chemotherapy, as seen with other axis like CXCR4/SDF-1 or VLA4/VCAM1. One hypothesis is that CCL2/CCR2 is only involved in migration as it occurs with normal leukocyte biology, since different concentrations and durations of co-culture of CCL2 did not induce chemotherapy protection. Also, the addition of other MCPs (MCP-2 and MCP-3) did not induce chemotherapy protection. Our hypothesis was that CCL2/CCR2 axis was related to chemotherapy protection as observed with other axis. However, our studies failed to demonstrate any sign of chemoprotection. Our hypothesis was that cell cycle modification was also involved in chemotherapy protection after inducing cell cycle arrest, but this too was not observed. Actually, we observed a significant increase in S phase and proliferation rate. Other models have shown that this axis is involved in proliferation and tumor progression [35–37]. We hypothesize that the lack of protection from the CCL2/CCR2 axis *in vitro* possibly relates to the fact that CCL2/CCR2 axis has to be connected *in vivo* to other axis like CXCR4/SDF-1 or VLA-4/VCAM1.

To show this, first we sought to study in an *in vivo* model if after CCR2 blockade, CCL2/CCR2 axis was involved in chemotherapy protection. In order to show this, we reproduced a previous model of AML mobilization to expose AML cells from their niche and make them sensitive to chemotherapy [3]. Overall, we did not observe any significant effect on normal HCS or AML mobilization and chemotherapy protection.

Recent evidence suggests that CCL2/CCR2 axis may possibly be involved in AML resistance to chemotherapy, not related to its role in AML migration and proliferation kinetics but related to subsets of immunosuppresive macrophages into the bone, which could be blocked with CCL2/CCR2 inhibitors and that way improve the response to chemotherapy [30]. Also, it has been suggested that other chemokines may be relevant in AML biology [38].

Finally, our results suggest that CCL2/CCR2 axis may have a role in a subset of AML patients, especially in monocytoid AML, which represent the leukemias with higher CCR2 expression. In normal monocytes its role is mostly related to cell migration. In our experiments its main role was related to migration and possibly to proliferation but not chemotherapy protection as we initially hypothesized, at least in the way the experiments were designed. It appears that other mechanisms for chemotherapy resistance may be involved including normal cells, like macrophages, as shown by recent evidence. However, multiple mechanisms involving not only the CCL2/CCR2 axis but as Brenner et al suggested [38], the chemokine milieu is important in AML biology. To targetone or several chemokines may affect AML susceptibility to chemotherapy and help develop new therapies for AML in the future.
